# Experiences of Black and White Family Hospice Caregivers: Anxiety, Depression, QOL, Burden, Hospice Communication

**DOI:** 10.1093/geroni/igab046.3150

**Published:** 2021-12-17

**Authors:** Lauren Starr, Karla Washington, Subhash Aryal, Debra Parker Oliver, George Demiris

**Affiliations:** 1 University of Pennsylvania, Philadelphia, Pennsylvania, United States; 2 Washington University in St. Louis, St. Louis, Missouri, United States; 3 University of Pennsylvania School of Nursing, Philadelphia, Pennsylvania, United States; 4 School of Nursing, University of Pennsylvania, University of Pennsylvania, Pennsylvania, United States

## Abstract

Although hospice care benefits seriously ill patients and their families, growing evidence suggests anxiety, depression, and altered quality of life are prevalent among family hospice caregivers. It is unknown if Black and white family hospice caregivers experience differences in mental health, quality of life, caregiver burden, or quality of hospice communication. In this secondary analysis of baseline data collected from 717 family hospice caregivers in two randomized clinical trials, we compared anxiety (GAD-7), depression (PHQ-9), quality of life (CQLI-R), caregiver burden (Zarit), and caregiver-reported quality of hospice team communication (CCCQ) between Black and white caregivers. Black and white caregivers differed demographically across multiple variables. In bivariate analysis, we found no differences in depression (P=0.3536), anxiety (P=0.0733), caregiver burden (P=0.6680), and perceptions of caregiver-centered hospice communication (P=0.4549). White caregivers reported lower quality of life than Blacks (P=0.0386), specifically in emotional (P=0.0321) and social (P=0.0002) domains. Financial and physical quality of life did not differ. In multivariate regression analyses controlling for caregiver and patient factors, we found no racial differences in depression (P= 0.5071), anxiety (P = 0.7288), quality of life (P=0.0584), caregiver burden (P=0.9465), or hospice communication (P=0.8779). Variables explained 7.7% to 20% of variability in outcomes, suggesting research is needed to understand which other factors contribute to hospice caregiver coping and communication experiences. Results suggest Black and white informal hospice caregivers experience similar levels of anxiety, depression, burden, and perceptions of hospice team communication quality. Interventions to support hospice caregivers across racial groups are needed.

